# Virtual Reality in the Pediatric Intensive Care Unit: Patient Emotional and Physiologic Responses

**DOI:** 10.3389/fdgth.2022.867961

**Published:** 2022-03-28

**Authors:** Colleen M. Badke, Sheila Krogh-Jespersen, Rachel M. Flynn, Avani Shukla, Bonnie S. Essner, Marcelo R. Malakooti

**Affiliations:** ^1^Division of Critical Care, Department of Pediatrics, Northwestern University Feinberg School of Medicine, Chicago, IL, United States; ^2^Pediatric Intensive Care Unit, Ann & Robert H. Lurie Children's Hospital of Chicago, Chicago, IL, United States; ^3^IGNITE Innovation, Ann & Robert H. Lurie Children's Hospital of Chicago, Chicago, IL, United States; ^4^Department of Medical Social Sciences, Feinberg School of Medicine, Northwestern University, Chicago, IL, United States; ^5^Institute for Innovations in Developmental Sciences, Northwestern University, Chicago, IL, United States; ^6^Department of Psychiatry and Behavioral Sciences, Northwestern University Feinberg School of Medicine, Chicago, IL, United States; ^7^Pritzker Department of Psychiatry and Behavioral Health, Ann & Robert H. Lurie Children's Hospital of Chicago, Chicago, IL, United States; ^8^Division of Gastroenterology, Hepatology, and Nutrition, Ann & Robert H. Lurie Children's Hospital of Chicago, Chicago, IL, United States

**Keywords:** critical care, pediatrics, innovation, virtual reality, heart rate variability, engagement

## Abstract

**Context:**

Patients in the Pediatric Intensive Care Unit (PICU) are limited in their ability to engage in developmentally typical activity. Long-term hospitalization, especially with minimal interpersonal engagement, is associated with risk for delirium and delayed recovery. Virtual reality (VR) has growing evidence as a safe, efficacious, and acceptable intervention for pain and distress management in the context of uncomfortable healthcare procedures, and for enhancing engagement in, and improving outcomes of rehabilitation therapy.

**Hypothesis:**

Critically ill children may experience high levels of engagement and physiologic effects while engaging with VR.

**Methods and Models:**

This cross-sectional study of 3–17-year-old children admitted to a PICU used a VR headset to deliver 360-degree immersive experiences. This study had a mixed-method approach, including standardized behavioral coding, participant and parent surveys, and participant physiologic responses. Investigators noted comments the child made about VR, observed emotional responses, and documented an engagement score. To determine physiologic response to VR, integer heart rate variability (HRVi) was collected 30 min before, during, and 30 min after VR.

**Results:**

One hundred fifteen participants were enrolled from 6/18 to 10/19, and they interacted with VR for a median of 10 min (interquartile range 7–17). Most children enjoyed the experience; 83% of participants smiled and 36% laughed while using VR. Seventy-two percent made positive comments while using VR. The strongest age-related pattern regarding comments was that the youngest children were more likely to share the experience with others. Seventy-nine percent of participants were highly engaged with VR. Ninety-two percent of parents reported that VR calmed their child, and 78% of participants felt that VR was calming. *HRVi Minimum* scores were significantly higher during VR than pre- (*p* < 0.001) or post-VR (*p* < 0.001). There was no significant difference between pre-and post-VR (*p* = 0.387); therefore, children returned to their pre-intervention state following VR.

**Interpretations and Conclusions:**

Children admitted to the PICU are highly engaged with and consistently enjoyed using VR. Both participants and parents found VR to be calming, consistent with intra-intervention physiologic improvements in HRVi. VR is an immersive tool that can augment the hospital environment for children.

## Introduction

Critically ill patients in the Pediatric Intensive Care Unit (PICU) are limited in their ability to engage in developmentally typical activity and may spend their days as passive observers of screens, such as television (1, 2). Long-term hospitalization, especially with minimal interpersonal engagement, is associated with risk of delirium and delayed recovery, often defined as post-intensive care syndrome (PICS) (3, 4). Incidence of these co-morbidities is increased in the PICU (3, 5), negatively impacts patient and family quality of life (4, 6), and may lead to short and long term cognitive or psychological impairment (4). Virtual reality (VR) technology has growing evidence as a safe, efficacious, and acceptable intervention for pain and distress management in the context of uncomfortable healthcare procedures, and for enhancing engagement in and improving outcomes of rehabilitation therapy. Our group recently demonstrated that VR is a safe, feasible, and innovative experience for critically ill children, and it is perceived as enjoyable and calming by both parents and patients (7). There is value in investigating VR for its potential in mitigating the negative morbidities of extended PICU admission.

Research on the potential mechanisms for how VR may benefit critically ill children is lacking. If VR subjectively improves a patient's response to a stressful or painful event (8, 9), this subjective experience is likely associated with a consistent, corresponding shift in physiologic response during, and potentially persisting past, the VR episode. Identifying a link between observed and perceived benefits of VR with physiologic biomarkers of health could reveal valuable clues to optimizing VR technology that is both subjectively satisfactory to patients and families and objectively beneficial for clinical health outcomes. However, it is currently unclear to what extent VR alters physiology. One way physiology can be measured at the bedside of a critically ill child is *via* heart rate variability (HRV), which is controlled by the autonomic nervous system (ANS). The ANS is a unique system that regulates functions in all organ systems, maintaining homeostasis when confronted with stressors, including severe infections (10). ANS dysfunction (ANSD) occurs during an imbalanced or maladaptive response to stress and is often due to excessive, uncontrolled, or prolonged sympathetic activation or inappropriate regulation by the parasympathetic nervous system (11). Most of the evidence on ANSD in critical illness is focused on HRV, a non-invasive, continuous physiologic marker of ANS function; low HRV is inversely correlated with organ dysfunction and mortality in adult and pediatric patients (12–15). Importantly, HRV also increases as critical illness resolves (16). It is unknown whether virtual reality has the potential to influence the ANS or modify the trajectory and recovery of HRV toward a child's baseline.

This study explores the emotional and physiological responses of critically ill children experiencing VR. The first objective was to explore whether children engaged in and enjoyed the VR experience through in-moment behavioral coding and parent- and participant-surveys. This includes whether there were any adverse effects of the experience. The second objective was to assess patterns of physiological responses by measuring changes in HRV associated with the VR experience.

## Materials and Methods

### Study Design and Patient Population

This cross-sectional, single institution study enrolled a convenience sample of 115 PICU patients. Eligible patients were 3-to 17-years-old, sufficiently alert to interact with VR, and capable of wearing the VR device (e.g., no obstructive medical equipment). Exclusion criteria included moderate or heavy sedation, vasoactive support, encephalopathy, recent neurologic injury, significantly impaired vision, or clinical instability. Mechanical ventilation was not an exclusion criterion, although given that many patients receiving mechanical ventilation also received moderate or heavy sedation, many of these patients were excluded. Informed consent was obtained prior to participation. This study was approved by Ann & Robert H. Lurie Children's Hospital of Chicago Institutional Review Board (IRB 2018-1564).

Given the large range in ages for this sample (3–17 years), the primary analyses focused on overall patterns for the full sample. As exploratory analyses, subgroups were generated in 3-year bands (*n* = 23 in each band) to examine whether there were developmentally based differences in how children experienced VR.

### VR Intervention

Three hundred-sixty-degree monoscopically recorded immersions were delivered using a simple VR headset and smartphone videos from a widely available multimedia source. This type of VR experience has been safely used in other research on pediatric patients under the age of 13 (7). Each participant selected a set of curated, developmentally appropriate VR experiences organized into themes of adventure (e.g., snowboarding and roller coasters), animals (e.g., puppies, bunnies, and lions), or nature (e.g., serene landscapes). A parent or investigator chose videos in cases in which the child had challenges making the decision. The research investigator monitored participants for anxiety or distress (e.g., child comments or body language indicating dissatisfaction) and recorded any adverse events. Children were permitted to use VR for as long as they requested and the duration of the VR experience was recorded by investigators.

### Behavioral Observations: Participant Verbal and Behavioral Indicators of Engagement With VR

During the session, the research investigator documented three categories of behavioral observation data points through written field notes. The first set of data collected was the content of child comments while using VR, which were later categorized according to: (1) emotional expression, including “positively valenced” or “negatively valenced” categories; and (2) content of participant utterances. Open-ended comments were coded into thematic categories and age-related differences in the types of comments being made were examined with a multivariate ANOVA. The second set of data collected was the total of number of smiles and laughs the child demonstrated while using VR. These behaviors were chosen for the high reliability at which coders could consistently identify them and their unambiguous fit as behavioral indicators of positive emotions in this context. The third set of data collected was an “engagement score”. This investigator-developed score ranked the child's engagement on a 10-point scale, referring to three categories, with scores of 8–10 classified as “total distraction” (i.e., highest degree of engagement in the VR experience), 4–7 “partial distraction” (i.e., moderate engagement), and 1–4 being “simple distraction” (i.e., lowest degree of engagement). The two investigators who conducted the majority of the VR events co-observed 24 encounters (21%).

### Parent- and Child Participant-Report: VR Acceptability and Subjective Emotional Response

Immediately following VR, parents and participants completed an investigator-developed questionnaire (Supplementary Figure 1). On a 4-point Likert scale (1 = strongly agree, 4 = strongly disagree), parents were asked if their child enjoyed VR, if they enjoyed watching their child use VR, if their child wanted to use VR longer, if VR calmed their child, and if VR was confusing, difficult, or uncomfortable. Parents were also asked about prior VR use, observed adverse effects, and if they would allow future use of VR. Parents responded with Yes (1) or No (0) to these questions. The participant survey asked similar questions, using the same Likert scale (Supplementary Figure 2). The surveys concluded with open-ended feedback about the VR experiences.

### Heart Rate Variability Measurement

Continuous heart rate data were extracted from bedside monitors using the BedMaster system (Hillrom, Jupiter, FL) and stored locally in an archiving system within hospital servers. HRV was estimated using the integer HRV (HRVi), which was calculated as the standard deviation of the heart rate sampled every 1 s over 5 consecutive minutes and normalized for age as previously described (15). A minimum of 50% of the heart rate samples in each 5-min interval were needed to be included in the analysis. HRVi was assessed at three time points: occurring 30 min before the VR experience started (“pre”), during VR (“during”), and 30 min after the VR experience ended (“post”). The minimum and median HRVi values were calculated for each of these time periods. We assessed for main effects of time using an Analysis of Variance (ANOVA). We also used a series of Analysis of Covariance (ANCOVA) tests to examine interaction effects between HRVi, time, and the following covariates, sex, age, ethnicity, type of VR experience the child chose, duration of VR encounter, length of stay in the PICU and level of severity of the illness.

### Participant Demographic and Clinical Information

Participant demographic information and clinical characteristics were collected *via* chart review. One hundred fifteen participants were enrolled from June 2018 through October 2019. Participant characteristics are reported in Table 1. The median age was 10 years [interquartile range (IQR) 6–13]. Forty-nine percent of participants were male and 29% identified as Hispanic/Latino. The most common reason for PICU admission was respiratory disease (34%). VR was administered most often on the first day of PICU admission (median 0.8 days, IQR 0.2–2.4). Participants had a median PICU length of stay of 2.7 days [interquartile range (IQR) 1.5–4.8] and hospital length of stay of 6.3 days (IQR 3.1–17.3). The median pediatric risk of mortality (PRISM) III score, a marker of illness severity on admission, was 3.0 (IQR 0–7) (Table 1).

**Table 1:** Demographic and clinical characteristics of study population (*N* = 115).

**Characteristic**	***N*** **(%)**
Age (year), median (IQR)	*M* = 10 (6–13)
**Sex**	
Male	56 (48.7%)
**Ethnicity**	
Hispanic/Latino	33 (28.7%)
**Primary PICU diagnosis**	
Respiratory disease	39 (33.9%)
Post-surgical	26 (22.6%)
Neurologic disease	13 (11.3%)
Shock/sepsis	18 (15.7%)
Other	19 (16.5%)
Participants who used VR in the past	33 (28.7%)
PICU admission day at time of VR (day), median (IQR)	0.8 (0.2–2.4)
PICU length of stay (median, IQR)	2.7 (1.5–4.8)
Hospital length of stay (median, IQR)	6.3 (3.1–17.3)
PRISM^*^ III score (median, IQR)	3.0 (0–7)

* *Pediatric Risk of Mortality*.

## Results

### Behavioral Observations

The majority of patients selected the video category of “animals” (51%), followed by adventure (35%) and nature (11%). Children enjoyed the experience (Table 2); 83% percent of participants smiled while using VR, and 36% laughed while using VR. Seventy-two percent of participants made positively valenced comments while using VR (e.g., “This is really cool,” “This is so awesome,” “Oh my gosh!” and “Whoa!”), with only 9% of participants sharing negatively valenced comments during the experience. The median engagement score was 9 (IQR 8–10), with 91 children (79%) rated as “total distraction” (i.e., highest degree of engagement) (Supplementary Table 1). Inter-rater reliability for the engagement in VR score was modest, with percent agreement for engagement 77.3%.

**Table 2:** Participant and parent survey responses (*N* = 112).

	**Survey question**	***N*** **(%) Strongly agree**	***N*** **(%) Agree**	***N*** **(%) Disagree**	***N*** **(%) Strongly disagree**
Participant responses	1. I enjoyed using virtual reality	58 (50%)	51 (44%)	3 (3%)	0 (0%)
	2. I wanted to use virtual reality for a longer period of time	18 (16%)	52 (45%)	40 (35%)	2 (2%)
	3. Virtual reality was calming	28 (24%)	62 (54%)	15 (13%)	2 (2%)
	4. Virtual reality was confusing or difficult	3 (3%)	10 (9%)	73 (63%)	26 (23%)
	5. Virtual reality was uncomfortable	2 (2%)	25 (22%)	66 (57%)	19 (17%)
Parent responses	1. My child enjoyed using virtual reality	66 (57%)	44 (38%)	2 (2%)	0 (0%)
	2. I enjoyed watching my child use VR	61 (53%)	50 (43%)	1 (0%)	0 (0%)
	3. My child wanted to use virtual reality for a longer period of time	26 (23%)	48 (42%)	35 (30%)	2 (2%)
	4. Virtual reality calmed my child	34 (30%)	68 (59%)	7 (6%)	2 (2%)
	5. Virtual reality was confusing or distracting	0 (0%)	10 (9%)	53 (55%)	38 (33%)
	6. Virtual reality was uncomfortable for my child	1 (1%)	12 (10%)	59 (51%)	40 (35%)

### Parent- and Child Participant-Report

Ninety-seven percent of participants and parents completed the post-VR survey (Table 2). Ninety-seven percent of participants agreed or strongly agreed that they enjoyed using VR, and 68% wanted to use VR for a longer duration. Ninety-eight percent of parents agreed or strongly agreed that their child enjoyed VR, and 99% agreed or strongly agreed that they enjoyed watching their child use VR. Ninety-two percent of parents reported that VR calmed their child, and 78% of participants felt that VR was calming. Longer duration of the VR encounter was significantly associated with participant and parent reports of greater child enjoyment of the VR experience [child: *r*_(112)_ = 0.37, *p* < 0.001; parent: *r*_(112)_ = 0.28, *p* = 0.006]. Similarly, there was a positive association between greater length of VR encounter and higher parent- and child-reported rating of VR as calming [child: *r*_(108)_ = 0.27, *p* = 0.005; parent: *r*_(111)_ = 0.28, *p* = 0.003].

### Exploratory Age-Related Analyses

#### Behavioral Observations

Patients interacted with VR for a median of 10 min (*M* = 12 min; IQR 7–17). There were no age-related differences in the length of viewing by age group [*F*_(4, 114)_ = 0.36, *p* = 0.84]. In some circumstances, parents or the researchers selected the category of video, which occurred more frequently for child participants in the 3–5 year-old group (44%) than older ages groups (6–8 years: 30%; 9–11 years: 26%; 12–14 years: 8%; 15–17 years: 0%). When the child selected the video, the youngest age groups preferred “animals” (3–5 years: 83%; 6–8 years: 63%), whereas the older age groups preferred “adventure” (9–11 years: 65%; 12–14 years: 52%; 15–17 years: 48%). There were no significant differences across ages in the total number of positively or negatively valenced comments during VR encounter, although the number of positive comments was trending toward significance [*F*_(4, 110)_ = 2.12, *p* = 0.083]. *Post-hoc* comparisons with Fisher's Least Significant Difference (LSD) test revealed that this was driven primarily by low numbers of positive responses in the 3–5 year-old group and 12–14 year-old group compared to other groups (Supplementary Table 2). There were no significant age group differences in the number of smiles or laughter occurrences.

#### Parent- and Child Participant-Report

Open-ended comments were coded into the following categories: Blurring the real and virtual worlds [e.g., “Wow how are you [monkey] up there?”, “Can my bunny come to life?”, and “Aww he is talking to me”; *F*_(4, 114)_ = 2.08, *p* = 0.088]; general experiences with the device [e.g., “I want to turn around” and “Too dizzy, fun dizzy though”; *F*_(4, 114)_ = 2.59, *p* = 0.041]; descriptions of the video content [e.g., “It's a beautiful mountain,” “The lion sneezed on me,” “I see a puppy,” and “It's SpongeBob haha”; *F*_(4, 114)_ = 2.58, *p* = 0.041]; descriptions related to time and space [e.g., “There is a plane on top of my head,” “We're upside down,” and “I am going to float over that cliff”; *F*_(4, 114)_ = 2.87, *p* = 0.027]; and sharing the experiences with others in the room [e.g., “Whoa mom, look at this”; *F*_(4, 114)_ = 3.40, *p* = 0.012]. Although varied age patterns are evident (see Supplementary Table 3 for means and standard deviations; Supplementary Tables 4–8 for significance between ages), generally, the strongest age-related pattern regarding comments was that the youngest children were more likely to attempt to share the experience with people in the room than older children.

### Adverse Events

Ten children (8%) made comments indicating that they experienced mild discomfort during the VR experience, but none required or requested discontinuation of their encounter. Five children (4%) commented that they felt dizzy or nauseated (i.e., motion sick). Two children (2%) commented on vision-related discomfort. One child started coughing (not VR related), one had neck discomfort, and one adverse event was not further specified.

### Heart Rate Variability Results

#### HRVi Median

**Figure 1:**
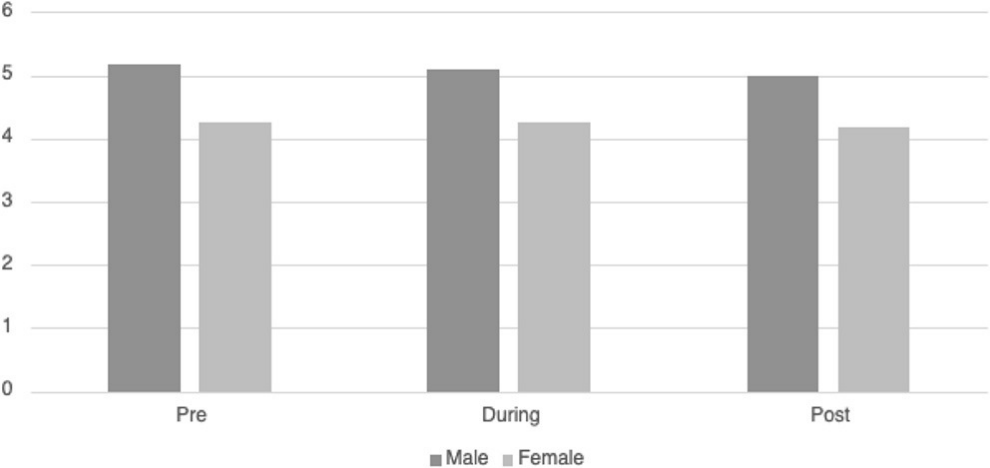
Differences in *HRVi Median* by sex. Males had higher HRVi Median at every time point compared to females (*F*(2, 93) = 5.45, *p* = 0.022, η^2^*p* = 0.055).

Ninety-five patients had sufficient continuous heart rate data for HRVi analysis. Table 3 displays the descriptive information for *HRVi Median* pre-, during, and post-VR experience. There was no significant differences in *HRVi Median* scores at the different time points (*p* = 0.73). There was a small main effect of sex [*F*_(2, 93)_ = 5.45, *p* = 0.022, η^2^_*p*_ = 0.055] by which males had higher *HRVi Median* at every time point compared to females (see Figure 1). There was not a main effect of time or an interaction effect between sex and time. There were no main effects or interaction effects with age, ethnicity, the type of VR experience children chose, duration of VR encounter, length of stay in the PICU, or level of severity of illness (all *p's* > 0.10).

**Table 3:** HRVi median and minimum pre-, during and post-VR.

	**Pre-**	**During**	**Post-**
**HRVi Median**			
*N*	95	95	95
Mean (SD)	4.67 (1.89)	4.64 (2.89)	4.56 (1.83)
Range	1.48–9.63	0.92–12.84	1.65–9.65
Median	4.47	4.58	4.44
**HRVi Minimum**			
*N*	95	95	95
Mean (SD)	2.88 (1.48)	4.01 (2.22)	2.78 (1.21)
Range	0.82–8.45	0.84–12.84	0.65–6.52
Median	2.43	3.49	2.73

#### HRVI Minimum

**Figure 2:**
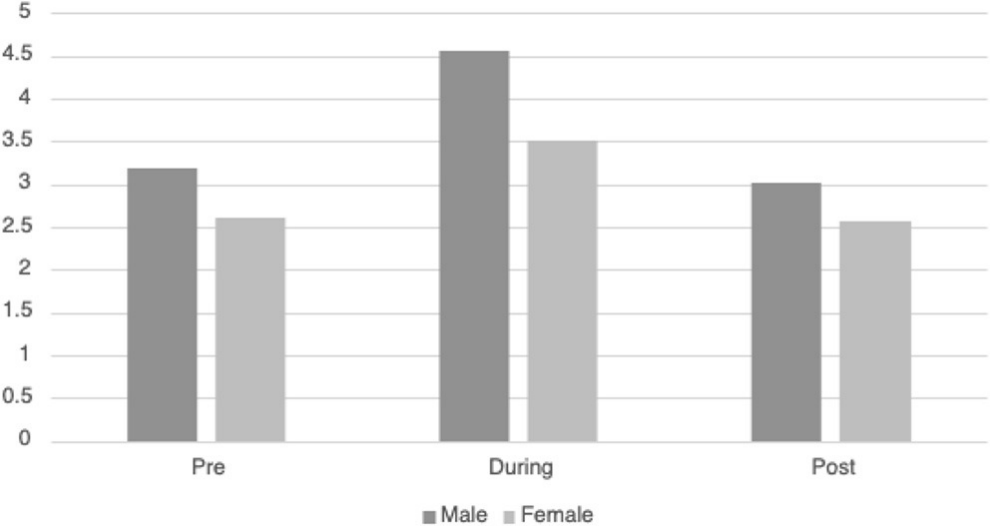
Differences in *HRVi Minimum* by sex. Males had a higher *HRVi Minimum* at every time point compared to females (*F*(2, 93) = 34.011, *p* = 0.015, η^2^*p* = 0.062).

**Figure 3:**
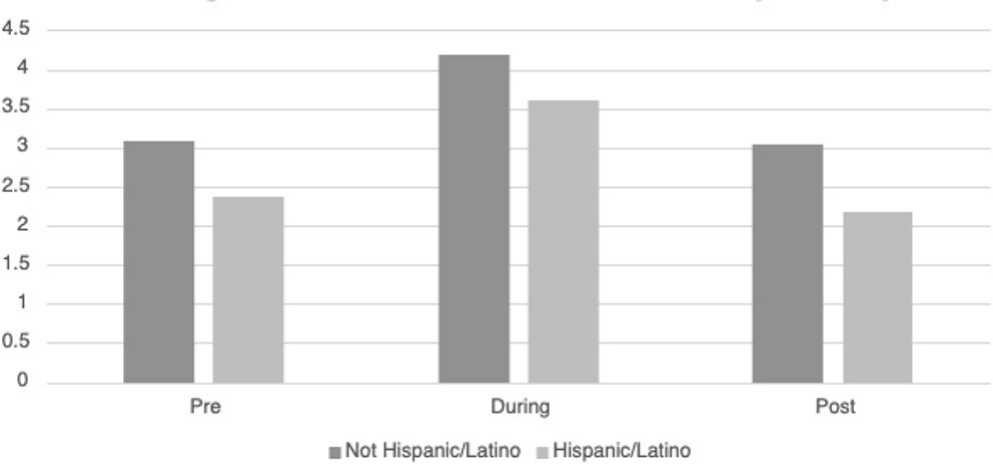
Differences in *HRVi Minimum* by ethnicity. Children who were not Hispanic/Latino had higher *HRVi Minimum* at every time point compared to children who were Hispanic/Latino (*F*(2, 93) = 5.27, *p* = 0.024, η^2^*p* = 0.054).

Table 3 displays the descriptive information for *HRVi Minimum* pre-, during, and post-VR experience. There was a significant quadratic relationship for *HRVi Minimum* scores, *F*_(2, 93)_ = 32.24, *p* < 0.001, η^2^_*p*_ = 0.255. *Post-hoc* analysis revealed that children's *HRVi Minimum* scores were significantly higher during the VR experience than pre- (*p* < 0.001) or post-VR (*p* < 0.001). There was no significant difference between pre- and post-VR (*p* = 0.387); therefore, children returned to their state after playing. There was a small main effect of sex [*F*_(2, 93)_ = 34.011, *p* = 0.015, η^2^_*p*_ = 0.062] where males had a higher *HRVi Minimum* at every time point compared to females (see Figure 2); the main effect of time remained significant in this model, but there was not an interaction effect. There was a small main effect of ethnicity [*F*_(2, 93)_ = 5.27 *p* = 0.024, η^2^_*p*_ = 0.054] where children who were not Hispanic/Latino had higher *HRVi Minimum* at every time point compared to children who were Hispanic/Latino (see Figure 3), the main effect of time remained significant in this model, but there was not an interaction effect. There was a significant interaction with duration of VR encounter [*F*_(2, 93)_ = 6.5, *p* = 0.002, η^2^_*p*_ = 0.065] and a main effect of duration [*F*_(2, 93)_ = 5.83, *p* = 0.018, η^2^_*p*_ = 0.059]. Duration was correlated with *HRVi Minimum* only during the VR experience, *r* = −0.307, *p* = 0.002; the longer a child used VR the lower their *HRVi Minimum* score was. There were no main effects or interaction effects with age, the type of VR experience children chose, length of stay in the PICU, or level of severity of illness (all *p's* > 0.20).

## Discussion

The current study demonstrates the practicability of deploying VR for critically ill children hospitalized in the PICU. There were a few adverse events that were all mild, consistent with prior literature (17). Additionally, children demonstrated high engagement, and both children and parents reported that they enjoyed the experience. Indeed, longer periods of engagement were associated with higher child- and parent-reports of enjoyment and calming; overall, child participants and parents described VR to be a subjectively calming experience. Across all age groups, participants made few to no negatively valenced comments during the VR encounter, and positive comments explicate how children through adolescents perceived the VR world. Interestingly, participants of all ages had a positive experience with VR. Age patterns were identified related to child comments about the VR experience, with the strongest pattern being that younger children were more likely than older children to share the experience with people in the room. Lastly, we found brief physiologic changes in HRVi measurements for participants while using VR, which were not sustained in the period after VR. This suggests that the physiologic impact of VR on longer-term PICU outcomes, such as PICS, may be lacking, but more research is needed.

By employing auditory, visual, and sometimes tactile stimulation, VR distracts participants from the “real world.” It can be applied in many settings, including inpatient medical settings (18–20). In pediatrics, including in critically ill children, VR is a safe, cost-effective, efficacious, and acceptable intervention for managing acute, procedure-related pain and emotional distress (7, 19–25). Our study supports these findings; in this cohort of critically ill children, we found very high levels of satisfaction and engagement with VR, with low adverse events. Additional uses for VR in the pediatric inpatient setting include managing pain and anxiety (8), such as its use as a distraction tool in burn care (9). It can also be employed as an adjunct to motor and sensory deficit rehabilitation (26, 27). Future work should examine the efficacy of VR in critically ill children undergoing procedures or rehabilitation and examine the potential impact on critically ill children who are at risk for delirium or PICS.

The physiologic mechanisms behind improving anxiety, pain, and distraction remain unclear. Our work shows that there are brief changes in HRVi while using VR, but these are not sustained. When HRV has been examined in other sympathetic-driven activities such as exercise, both heart rate and HRV change during the activity then demonstrate a time-dependent recovery, with a rapid (yet incomplete) recovery to pre-exercise levels, and a complete recovery within 48 h (28). Additionally, the effect of exercise on HRV is mediated by the intensity of exercise, with results also demonstrating an association between the duration of exercise and HRV (29). We found that VR duration correlated with the minimum HRVi, with a time-dependent recovery within the 30 min post-VR. Interestingly, the median HRVi did not improve in this setting. We hypothesize that the minimum HRVi may have increased due to the calming nature of VR affecting parasympathetic output, however the median was statistically unaffected due to the pervasive imbalance between sympathetic and parasympathetic output. It is unclear if more frequent use, longer duration, or more intense VR experience would result in a more sustained balance between sympathetic and parasympathetic output and a longer time-to-recovery. While this study did not demonstrate a lasting improvement in HRV after the VR intervention, we acknowledge that a single administration of VR is likely insufficient to sustain an impact significant enough to outweigh ICU-related co-morbidities of hospitalization. Given that HRV increases as critical illness resolves (16), it remains unknown whether VR can influence the trajectory of HRV recovery in critically ill children.

This study has several strengths and limitations. Although this was a large sample size of critically ill children, being a single center study may have limited generalizability. Many children were recovering from critical illness at the time of enrollment and may have already returned to their baseline HRV; future work could examine the impact of VR on the physiology of children with more severe critical illness. We were unable to account for exposure to medications that could influence the autonomic nervous system, such as bronchodilators or sedatives. However, for children who were on these medications continuously, we would expect a blunted effect on HRVi during all three time points; therefore, the effect of VR on HRVi may be stronger when controlling for these potential confounders. Additionally, we administered VR for only one session per patient, thereby limiting our ability to assess for a dose-response relationship with PICU outcomes. A sustained physiologic response and impact on longer term outcomes such as PICS might be observed if there is a dose-response relationship. Next, we employed commercial VR products that lack diversity and meaningful design strategies; this may have limited engagement and physiologic response beyond simple distraction techniques (8). Furthermore, we calculated HRVi using integer heart rate data, which only requires a lightweight implementation and may be more generalizable than the more computationally-intense derivation of HRV from R-R intervals in the electrocardiogram waveform. However, HRVi may not completely capture the complexity of physiologic response in this population. Finally, throughout the study period there were data losses due to technical issues, most commonly server downtimes without back-ups. We do not believe this represents systematic bias in the study population as these data losses occurred at random.

In conclusion, children admitted to the PICU are highly engaged with and consistently enjoy using VR. Both parents and participants find VR to be a calming experience, which may be supported by brief physiologic changes in heart rate variability during the VR experience. VR is a tool that can be used to augment the hospital environment, and further research is required to understand the potential impact on patient physiology and clinical outcomes.

## Data Availability Statement

The raw data supporting the conclusions of this article will be made available by the authors, without undue reservation.

## Ethics Statement

The studies involving human participants were reviewed and approved by Ann & Robert H. Lurie Children's Hospital of Chicago. Written informed consent to participate in this study was provided by the participants' legal guardian/next of kin.

## Author Contributions

CB and MM designed the study. CB, AS, and MM performed data collection. CB, SK-J, and RF performed data analysis. All authors made substantial contributions to drafting and final approval of the manuscript and agree to be accountable for the content of the work.

## Conflict of Interest

The authors declare that the research was conducted in the absence of any commercial or financial relationships that could be construed as a potential conflict of interest.

## Publisher's Note

All claims expressed in this article are solely those of the authors and do not necessarily represent those of their affiliated organizations, or those of the publisher, the editors and the reviewers. Any product that may be evaluated in this article, or claim that may be made by its manufacturer, is not guaranteed or endorsed by the publisher.

## Abbreviations

ANSD: autonomic nervous system dysfunction
HRV: heart rate variability
HRVi: integer heart rate variability
PICS: post-intensive care syndrome
PICU: pediatric intensive care unit
VR: virtual reality.

## References

[1] Sood E Berends WM Butcher JL Lisanti AJ Medoff-Cooper B Singer J. Developmental care in North American pediatric cardiac intensive care units: survey of current practices. Adv Neonatal Care. (2016) 16:211–9. 10.1097/ANC.0000000000000264 27140031 PMC5659348

[2] Garcia Guerra G Joffe AR Cave D Duff J Duncan S Sheppard C. Survey of sedation and analgesia practice among canadian pediatric critical care physicians. Pediatr Crit Care Med. (2016) 17:823–30. 10.1097/PCC.0000000000000864 27467012

[3] Rawal G Yadav S Kumar R. Post-intensive care syndrome: an overview. J Transl Int Med. (2017) 5:90–2. 10.1515/jtim-2016-0016 28721340 PMC5506407

[4] Watson RS Choong K Colville G Crow S Dervan LA Hopkins RO. Life after critical illness in children-toward an understanding of pediatric post-intensive care syndrome. J Pediatr. (2018) 198:16–24. 10.1016/j.jpeds.2017.12.084 29728304

[5] Hopkins RO Choong K Zebuhr CA Kudchadkar SR. Transforming picu culture to facilitate early rehabilitation. J Pediatr Intensive Care. (2015) 4:204–11. 10.1055/s-0035-1563547 27134761 PMC4849412

[6] Petrinec AB Martin BR. Post-intensive care syndrome symptoms and health-related quality of life in family decision-makers of critically ill patients. Palliat Supp Care. (2018) 16:719–24. 10.1017/S1478951517001043 29277171

[7] Badke CM Essner BS O'Connell M Malakooti MR. An innovative virtual reality experience in the picu: a pilot study. Pediatr Crit Care Med. (2019) 20:e283–e6. 10.1097/PCC.0000000000001917 30920437

[8] Ahmadpour N Keep M Janssen A Rouf AS Marthick M. Design strategies for virtual reality interventions for managing pain and anxiety in children and adolescents: scoping review. JMIR Serious Games. (2020) 8:e14565. 10.2196/14565 32012042 PMC7055787

[9] Eijlers R Utens E Staals LM de Nijs PFA Berghmans JM Wijnen RMH. Systematic review and meta-analysis of virtual reality in pediatrics: effects on pain and anxiety. Anesth Analg. (2019) 129:1344–53. 10.1213/ANE.0000000000004165 31136330 PMC6791566

[10] Badke CM Marsillio LE Weese-Mayer DE Sanchez-Pinto LN. Autonomic nervous system dysfunction in pediatric sepsis. Front Pediatr. (2018) 6:280. 10.3389/fped.2018.00280 30356758 PMC6189408

[11] Dunser MW Hasibeder WR. Sympathetic overstimulation during critical illness: adverse effects of adrenergic stress. J Intensive Care Med. (2009) 24:293–316. 10.1177/0885066609340519 19703817

[12] Ellenby MS McNames J Lai S McDonald BA Krieger D Sclabassi RJ. Uncoupling and recoupling of autonomic regulation of the heart beat in pediatric septic shock. Shock. (2001) 16:274–7. 10.1097/00024382-200116040-00007 11580109

[13] Goldstein B Fiser DH Kelly MM Mickelsen D Ruttimann U Pollack MM. Decomplexification in critical illness and injury: relationship between heart rate variability, severity of illness, and outcome. Crit Care Med. (1998) 26:352–7. 9468175 10.1097/00003246-199802000-00040

[14] Papaioannou VE Maglaveras N Houvarda I Antoniadou E Vretzakis G. Investigation of altered heart rate variability, nonlinear properties of heart rate signals, and organ dysfunction longitudinally over time in intensive care unit patients. J Crit Care. (2006) 21:95–103; discussion 4. 10.1016/j.jcrc.2005.12.007 16616632

[15] Badke CM Marsillio LE Carroll MS Weese-Mayer DE Sanchez-Pinto LN. Development of a heart rate variability risk score to predict organ dysfunction and death in critically ill children. Pediatr Crit Care Med. (2021) 22:e437–47. 10.1097/PCC.0000000000002707 33710071

[16] Marsillio LE Manghi T Carroll MS Balmert LC Wainwright MS. Heart rate variability as a marker of recovery from critical illness in children. PLoS ONE. (2019) 14:e0215930. 10.1371/journal.pone.0215930 31100075 PMC6524820

[17] Tychsen L Foeller P. Effects of immersive virtual reality headset viewing on young children: visuomotor function, postural stability, and motion sickness. Am J Ophthalmol. (2020) 209:151–9. 10.1016/j.ajo.2019.07.020 31377280

[18] Dascal J Reid M IsHak WW Spiegel B Recacho J Rosen B. Virtual reality and medical inpatients: a systematic review of randomized, controlled trials. Innov Clin Neurosci. (2017) 14:14–21. 28386517 PMC5373791

[19] Gold JI Mahrer NE. Is virtual reality ready for prime time in the medical space? A randomized control trial of pediatric virtual reality for acute procedural pain management. J Pediatr Psychol. (2018) 43:266–75. 10.1093/jpepsy/jsx129 29053848

[20] Agrawal AK Robertson S Litwin L Tringale E Treadwell M Hoppe C. Virtual reality as complementary pain therapy in hospitalized patients with sickle cell disease. Pediatric Blood Cancer. (2019) 66:e27525. 10.1002/pbc.27525 30362236

[21] Gershon J Zimand E Pickering M Rothbaum BO Hodges L. A pilot and feasibility study of virtual reality as a distraction for children with cancer. J Am Acad Child Adolesc Psychiatry. (2004) 43:1243–9. 10.1097/01.chi.0000135621.23145.05 15381891

[22] Das DA Grimmer KA Sparnon AL McRae SE Thomas BH. The efficacy of playing a virtual reality game in modulating pain for children with acute burn injuries: a randomized controlled trial [Isrctn87413556]. BMC Pediatr. (2005) 5:1. 10.1186/1471-2431-5-1 15745448 PMC554986

[23] Faber AW Patterson DR Bremer M. Repeated use of immersive virtual reality therapy to control pain during wound dressing changes in pediatric and adult burn patients. J Burn Care Res. (2013) 34:563–8. 10.1097/BCR.0b013e3182777904 23970314 PMC3770783

[24] Malloy KM Milling LS. The effectiveness of virtual reality distraction for pain reduction: a systematic review. Clin Psychol Rev. (2010) 30:1011–8. 10.1016/j.cpr.2010.07.001 20691523

[25] Li A Montano Z Chen VJ Gold JI. Virtual reality and pain management: current trends and future directions. Pain Manag. (2011) 1:147–57. 10.2217/pmt.10.15 21779307 PMC3138477

[26] Salem Y Elokda A. Use of virtual reality gaming systems for children who are critically ill. J Pediatr Rehabil Med. (2014) 7:273–6. 10.3233/PRM-140296 25260510

[27] Dunn J Yeo E Moghaddampour P Chau B Humbert S. Virtual and augmented reality in the treatment of phantom limb pain: a literature review. Neuro Rehabil. (2017) 40:595–601. 10.3233/NRE-171447 28211829

[28] Stanley J Peake JM Buchheit M. Cardiac parasympathetic reactivation following exercise: implications for training prescription. Sports Med. (2013) 43:1259–77. 10.1007/s40279-013-0083-4 23912805

[29] Michael S Graham KS Davis GMO. Cardiac autonomic responses during exercise and post-exercise recovery using heart rate variability and systolic time intervals-a review. Front Physiol. (2017) 8:301. 10.3389/fphys.2017.00301 28611675 PMC5447093

