# Supplementary material for: Virtual Reality in the Pediatric Intensive Care Unit: Patient Emotional and Physiologic Responses
Source: Front Digit Health. 2022 Mar 28;4:867961. doi: 10.3389/fdgth.2022.867961 (PMC8995472; doi:10.3389/fdgth.2022.867961)
Supplement: Supplementary file 1 [file Data_Sheet_1.pdf]

Supplementary Figure 1. Parent/Caregiver questionnaire

Virtual Reality in the PICU: Parent/Caregiver Survey

Please answer the following questions about your child:

1. What is your relationship to the child? Circle one:
   Mother Father Grandparent Aunt/Uncle Other: ________________
2. Has the child ever used virtual reality before? Circle one: Yes No

Please answer the following questions (circle your response):

3. The child enjoyed using virtual reality

   Strongly agree Agree Disagree Strongly Disagree

4. I enjoyed watching the child use virtual reality

   Strongly agree Agree Disagree Strongly Disagree

5. The child wanted to use virtual reality for a longer period of time

   Strongly agree Agree Disagree Strongly Disagree

6. The virtual reality experience calmed the child

   Strongly agree Agree Disagree Strongly Disagree

7. Virtual reality was confusing/difficult to use

   Strongly agree Agree Disagree Strongly Disagree

8. The virtual reality device was uncomfortable for the child

   Strongly agree Agree Disagree Strongly Disagree

9. Did the virtual reality experience cause any side effects or problems in the child?
   Circle one: Yes No
   a. If yes, please describe ____

10. Would you allow the child to use virtual reality in the future?
    Circle one: Yes No
    a. If no, please describe why not
    ____

Lastly, we would like to ask some questions about you:

11. How old are you? (Circle one)
    a. 18-25
    b. 26-30
    c. 31-35
    d. 36-40
    e. 41-45
    f. 46-50
    g. >50

12. Have you ever used virtual reality before? Circle one: Yes No

13. Do you have any suggestions for making the virtual reality experience better or easier to use?

____

____

____

____

Supplementary Figure 2. Participant questionnaire

Virtual Reality in the PICU: Patient Survey

Please circle your answer to the following responses:

1. Have you ever used virtual reality before? Yes No

2. I enjoyed using virtual reality

   Strongly agree Agree Disagree Strongly Disagree

3. I wanted to use virtual reality for a longer period of time

   Strongly agree Agree Disagree Strongly Disagree

4. The virtual reality experience was calming

   Strongly agree Agree Disagree Strongly Disagree

5. Virtual reality was confusing or difficult to use

   Strongly agree Agree Disagree Strongly Disagree

6. The virtual reality device was uncomfortable to wear

   Strongly agree Agree Disagree Strongly Disagree

7. Did the virtual reality experience cause any side effects or problems?
   Circle one: Yes No
   a. If yes, please describe ____

8. Do you want to use virtual reality in the future?
   Circle one: Yes No
   a. If no, please describe why not
      ____

9. Do you have any suggestions for making the virtual reality experience better or easier to use?

Supplementary Table 1. Quantitative VR experience from each child (N=115)

Characteristic	N (%)
Category of video watched	
Adventure	40 (35%)
Animals	59 (51%)
Nature	13 (11%)
Total time watching VR, minutes (median, IQR)	10 (7-17)
Participants who smiled	95 (82.6%)
Number of smiles per child (median, IQR)	3 (1-5)
Participants who laughed	41 (35.7%)
Number of smiles per child (median, IQR)	0 (0-2)
Participants with positive comment(s)	83 (72.2%)
Number of positive comments per child (median, IQR)	1.5 (0-4)
Participants with negative comment(s)	10 (8.7%)
Number of negative comments per child (median, IQR)	0 (0-0)
Engagement score (median, IQR)	9 (8-10)

Supplementary Table 2. Mean number of positive comments by age group

	Positive Comments		6-8 year olds	9-11 year olds	12-14 year olds	15-17 year olds
	M	*SD*				
3-5 year olds	1.96	2.65	2.11**	2.05**	0.18	0.48
6-8 year olds	4.11	3.89	-	0.07	2.3*	1.64
9-11 year olds	4.05	5.27		-	2.23*	1.57
12-14 year olds	1.82	2.8			-	0.66
15-17 year olds	2.48	3				-

* significant at .05 level

**trending < .07

Supplementary Table 3. Means and standard deviations for open-ended comments

	Blur Real & Virtual Worlds	VR Device	Video Content	Time & Space	Sharing the Experience
3-5 year olds	0.48 (0.79)	0.0 (0.0)	0.30 (0.47)	0.09 (0.29)	0.30 (0.47)
6-8 year olds	0.61 (0.84)	0.09 (0.29)	0.48 (0.51)	0.52 (0.90)	0.13 (0.34)
9-11 year olds	0.43 (0.66)	0.30 (0.56)	0.30 (0.47)	0.52 (0.90)	0.09 (0.29)
12-14 year olds	0.17 (0.39)	0.09 (0.42)	0.13 (0.34)	0.26 (0.62)	0.0 (0.0)
15-17 year olds	0.17(0.39)	0.04 (0.21)	0.13 (0.34)	0.04 (0.21)	0.02 (0.21)

Supplementary Table 4. Mean number of comments related to blurring the real and virtual worlds by age group

	Blur Real & Virtual Worlds Comments		6-8 year olds	9-11 year olds	12-14 year olds	15-17 year olds
	M	*SD*				
3-5 year olds	0.48	0.79	0.13	0.04	0.30	0.30
6-8 year olds	0.61	0.84	-	0.17	0.43*	0.43*
9-11 year olds	0.43	0.66	-	-	0.26	0.26
12-14 year olds	0.17	0.39	-	-	-	1.00
15-17 year olds	0.17	0.39	-	-	-	-

* significant at .05 level

Supplementary Table 5. Mean number of comments related to general experiences with the VR device by age group

	VR Device Comments		6-8 year olds	9-11 year olds	12-14 year olds	15-17 year olds
	M	*SD*				
3-5 year olds	0.0	0.0	0.09	0.30**	0.09	0.04
6-8 year olds	0.09	0.29	-	0.22*	0.0	0.04
9-11 year olds	0.30	0.56	-	-	0.22*	0.26*
12-14 year olds	0.09	0.42	-	-	-	0.04
15-17 year olds	0.04	0.21	-	-	-	-

* significant at .05 level
**significant at .01 level

Supplementary Table 6. Mean number of comments related to the video content by age group

	Video Content Comments		6-8 year olds	9-11 year olds	12-14 year olds	15-17 year olds
	M	*SD*				
3-5 year olds	0.30	0.47	0.17	0.0	0.17	0.17
6-8 year olds	0.48	0.51	-	0.17	0.35**	0.35**
9-11 year olds	0.30	0.47	-	-	0.17	0.17
12-14 year olds	0.13	0.34	-	-	-	0.0
15-17 year olds	0.13	0.34	-	-	-	-

* significant at .05 level

**significant at .01 level

Supplementary Table 7. Mean number of comments related to the time and space by age group

	Time & Space Comments		6-8 year olds	9-11 year olds	12-14 year olds	15-17 year olds
	M	*SD*				
3-5 year olds	0.09	0.29	0.43*	0.43*	0.17	0.04
6-8 year olds	0.52	0.90	-	0.0	0.26	0.48
9-11 year olds	0.52	0.90	-	-	0.26	0.48*
12-14 year olds	0.26	0.62	-	-	-	0.22
15-17 year olds	0.04	0.21	-	-	-	-

* significant at .05 level

Supplementary Table 8. Mean number of comments related to the sharing the experience by age group

	Sharing the Experience Comments		6-8 year olds	9-11 year olds	12-14 year olds	15-17 year olds
	M	*SD*				
3-5 year olds	0.30	0.47	0.17	0.22*	0.30**	0.26**
6-8 year olds	0.13	0.34	-	0.04	0.13	0.09
9-11 year olds	0.09	0.29	-	-	0.09	0.04
12-14 year olds	0.0	0.0	-	-	-	0.04
15-17 year olds	0.04	0.21	-	-	-	-

* significant at .05 level

**significant at .01 level
